# Assessment of Sensitivity and Specificity of Ultrasound and Magnetic Resonance Imaging in the Diagnosis of Placenta Accreta

**DOI:** 10.1055/s-0038-1675803

**Published:** 2018-11-14

**Authors:** Elisa Santos Lopes, Francisco Edson de Lucena Feitosa, Antonio Viana Brazil, José Daniel Vieira de Castro, Jesus Irajacy Fernandes da Costa, Edward Araujo Júnior, Alberto Borges Peixoto, Francisco Herlânio Costa Carvalho

**Affiliations:** 1 Department of Maternal and Child, Maternidade Escola Assis Chateaubriand, Universidade Federal do Ceará, Fortaleza, CE, Brazil; 2 Paulista School of Medicine, Department of Obstetrics, Universidade Federal de São Paulo, SP, Brazil; 3 Department of Obstetrics and Gynecology, Universidade Federal do Triângulo Mineiro, Uberaba, MG, Brazil; 4 Hospital Mário Palmério, Universidade de Uberaba, Uberaba, MG, Brazil

**Keywords:** placenta previa, placenta accreta, ultrasound, magnetic resonance imaging, placenta prévia, acretismo placentário, ultrassom, ressonância magnética

## Abstract

**Objective** To assess and compare the sensitivity and specificity of ultrasonography and magnetic resonance imaging in the diagnosis of placenta accreta in patients with placenta previa.

**Methods** This retrospective cohort study included 37 women, and was conducted between January 2013 and October 2015; 16 out of the 37 women suffered from placenta accreta. Histopathology was considered the gold standard for the diagnosis of placenta accreta; in its absence, a description of the intraoperative findings was used. The associations among the variables were investigated using the Pearson chi-squared test and the Mann-Whitney *U*-test.

**Results** The mean age of the patients was 31.8 ± 7.3 years, the mean number of pregnancies was 2.8 ± 1.1, the mean number of births was 1.4 ± 0.7, and the mean number of previous cesarean sections was 1.2 ± 0.8. Patients with placenta accreta had a higher frequency of history of cesarean section than those without it (63.6% versus 36.4% respectively; *p* < 0.001). The mean gestational age at birth among women diagnosed with placenta previa accreta was 35.4 ± 1.1 weeks. The mean birth weight was 2,635.9 ± 374.1 g. The sensitivity of the ultrasound was 87.5%, with a positive predictive value (PPV) of 65.1%, and a negative predictive value (NPV) of 75.0%. The sensitivity of the magnetic resonance imaging was 92.9%, with a PPV of 76.5%, and a NPV of 75.0%. The kappa coefficient of agreement between the 2 tests was 0.69 (95% confidence interval [95%CI]: (0.26–1.00).

**Conclusion** The ultrasound and the magnetic resonance imaging showed similar sensitivity and specificity for the diagnosis of placenta accreta.

## Abstract (pt)

**Resumo:**

**Objetivo** Avaliar e comparar a sensibilidade e especificidade da ultrassonografia e da ressonância magnética no diagnóstico do acretismo placentário em pacientes com placenta prévia.

**Métodos** Estudo de coorte retrospectivo com 37 mulheres, sendo 16 com acretismo placentário, realizado de janeiro de 2013 a outubro de 2015. Considerou-se padrão-ouro para o diagnóstico de acretismo placentário o exame anatomopatológico, sendo que, na sua ausência, a descrição do achado intraoperatório. As associações entre variáveis foram investigadas utilizando o teste qui-quadrado de Pearson e o teste *U* de Mann-Whitney.

**Resultados** A idade média foi de 31,8 ± 7,3 anos, o número médio de gestações foi de 2,8 ± 1,1, o número médio da quantidade de partos foi de 1,4 ± 0,7, e o número médio de cesáreas prévias foi de 1,2 ± 0,8. O grupo do acretismo placentário apresentou antecedente de cesariana mais frequentemente do que o grupo sem acretismo (63,6% versus 36,4%, respectivamente; *p* < 0,001). A idade gestacional no parto em mulheres com diagnóstico de placenta prévia com acretismo foi de 35,4 ± 1,1 semanas. O peso ao nascer médio foi de 2.635,9 ± 374,1 g. A sensibilidade do ultrassom foi de 87,5%, com valor preditivo positivo (VPP) de 65,1%, e valor preditivo negativo (VPN) de 75,0%. Para a ressonância magnética, a sensibilidade foi de 92,9%, com VPP de 76,5% e VPN de 75,0%. O índice kappa para concordância entre os dois testes foi de 0,69 (intervalo de confiança de 95% [IC95%]: 0,26–1,00).

**Conclusão** O ultrassom e a ressonância magnética apresentaram sensibilidade e especificidade semelhantes no diagnóstico do acretismo placentário.

## Introduction

Placenta accreta is characterized by an anomalous adherence of the placenta to the uterine wall. Based on the degree of adhesion, placental invasion can be classified into accreta, increta, or percreta.^1^ The global incidence of placenta accreta has been increasing over the years; this seems parallel to the increased rate of cesarean sections (C-sections). Wu et al^2^ reported the global incidence of placenta accreta as 1:533 pregnancies between 1982 and 2002, which is much higher than the incidences of 1:4,027 and 1:2,510 reported in the 1970s and 1980s.

Ultrasound (US) criteria are used to diagnose placental invasion, namely loss of the hypoechoic retroplacental myometrial zone, thinning or disruption of the uterine serosa–bladder interface, presence of exophytic zones and large sonolucent areas in the placenta, myometrial thickness < 1 mm, and, in Doppler US, turbulent flow of placental lacunae and bladder–uterine serosa interface hypervascularity.^3^ ^4^ ^5^

Magnetic resonance imaging (MRI) has been incorporated in the obstetric practice for some cases of fetal, maternal, and placental assessment in which soft tissues are clearly visible, thus enabling the assessment of the retroperitoneal space. The MRI does not seem to increase the possibility of diagnosing anterior placenta accreta; therefore, it is indicated for cases of posterior placenta, for which visualization by US is difficult owing to fetal parts.^6^

Few studies compared the accuracy of the MRI and US in the antenatal diagnosis of placental accretism, and similar prediction results have been found for both methods.^7^ ^8^ ^9^ ^10^ Satija et al^8^ concluded that the US remains the primary modality for the evaluation of placental accretism, and the MRI should be reserved for inconclusive cases. Rezk and Shawky^9^ compared Doppler US and MRI in patients with placenta previa and uterine scarring. Even though these authors did not make separate analyses for the placement of the placenta, they concluded that the MRI should be reserved to exclude accretion in cases of posterior or lateral placental location.^9^

The objective of the present study was to assess and compare the sensitivity and specificity of the US and MRI in the diagnosis of placenta accreta in patients with placenta previa.

## Methods

The present study is a retrospective cohort study of diagnostic assessment that was documented using data obtained from the records of patients hospitalized at the Department of Fetal Medicine, Maternidade Escola Assis Chateaubriand, Universidade Federal do Ceará, Fortaleza, Brazil, between January 2013 and October 2015. During this period, there was a total of 13,226 births; 7,009 (53.0%) of these were C-sections, and 6,217 (47%) were live births. The study protocol was analyzed and approved by the Ethics in Research Committee of Universidade Federal do Ceará (#1.471.764), with the need for informed consent waived owing to the study's retrospective nature.

The initial sample was composed of all 41 patients admitted to the institution during the study period with ultrasonographic diagnoses of placenta previa who had been examined due to complaints of transvaginal bleeding. All of those patients were submitted to at least one US examination, and, if possible, a single MRI examination. Four patients were excluded due to incomplete information. Data from the last US and MRI examinations performed on the last week before delivery were considered for the statistical analysis.

The US diagnosis of placenta previa was considered when the placental mass reached the internal cervical os. Cases of low placental insertion were not included in this series. There were 21 cases (56.8%) of total placenta previa, 12 (32.4%) of partial placenta previa, and 4 (10.8%) of marginal placenta previa. For the diagnosis of placenta accreta using Doppler US, we used at least 1 of the following criteria: 1) loss of the hypoechoic retroplacental myometrial zone; 2) disruption of the uterine serosa–bladder interface; 3) presence of exophytic zones; 4) large sonolucent areas in the placenta; 5) myometrial thickness < 1 mm; 6) turbulent blood flow in the placental lacuna; and 7) hypervascularity of theuterine serosa–bladder interface.^3^ ^4^ ^5^ For the diagnosis of placenta accreta by MRI, we used the following criteria: 1) presence of uterine bulging; 2) heterogeneous signal intensity within the placenta; 3) dark intraplacental bands on T2-weighted sequences,; 4) abnormal placental vascularity; 5) focal interruptions in the myometrial wall; 6) tenting of the bladder; and 7) direct visualization of the invasion of adjacent organs.^6^

The variables analyzed were: age; parity; number of C-sections; number of abortions; number of curettage procedures; type of delivery; and the need for hysterectomy. The placenta-related variables analyzed were: location of the placenta; and extent of placenta adherence. The newborn-related variables analyzed were: gestational age; birth weight; 1- and 5-minute Apgar scores; neonatal complications; and deaths. Histopathology was considered the gold standard for the diagnosis of placenta accreta; the description of the intraoperative findings was used in its absence.

Continuous variables were expressed as means and standard deviations, whereas nominal variables were expressed as absolute frequencies and percentages. The associations among the variables were investigated using the Pearson chi-squared test and the Mann-Whitney *U*-test. The Statistical Package for the Social Sciences (SPSS, IBM Corp., Armonk, NY, US) software, version 22.0, was used for all statistical analyses. Values of *p* < 0.05 were considered statistically significant.

## Results

Out of the 37 patients included in the study, 16 (43.2%) had placenta accreta, whereas 21 (56.8%) did not. One case (3%) progressed to vaginal birth, and 36 (97%), to C-section. Out of the 36 patients who had a C-section, 16 (44.4%) underwent concurrent hysterectomy, and 14 (87.5%) of these had placenta accreta confirmed by histopathology. Out of the 20 (55.6%) patients who had C-sections but no hysterectomy, 2 (10%) had placenta accreta confirmed by histopathology of the uterus segment that was resected or of the curettage material in the adhered segment.

Out of the 16 women with placenta accreta, 11 (68.8%) had anterior placentas, and 5 (31.2%) had posterior placentas. Regarding the degree of placental invasion, 9 (56.3%) women had placenta accreta, 6 (37.5%) had placenta increta, and 1 (6.2%) had placenta percreta. The mean age was 31.8 ± 7.3 years, the mean number of pregnancies was 2.8 ± 1.1, and the mean number of deliveries was 1.4 ± 0.7. The mean number of abortions was 0.3 ± 0.6, the mean number of curettage procedures was 0.4 ± 0.7, and the mean number of previous C-sections was 1.2 ± 0.8.

As for women without a diagnosis of placenta accreta, the mean age was 31 ± 6.8 years, the mean number of pregnancies was 3.5 ± 2.2, and the mean number of deliveries was 2 ± 2. The mean number of abortions was 0.5 ± 0.7, the mean number of curettage procedures was 0.5 ± 0.7, and the mean number of previous C-sections was 0.5 ± 0.7 (Table 1).

**Table 1:** Clinical and obstetric characteristics of the patients diagnosed with placenta previa with and without placenta accreta

Variable	With placenta accreta n = 16	Without placenta accreta n = 21	*p*-value*
Age (mean ± SD), years	31.8 ± 7.3	31 ± 6.8	0.774*
Pregnancies (mean ± SD)	2.8 ± 1.1	3.5 ± 2.2	0.387*
Deliveries (mean ± SD)	1.4 ± 0.7	2 ± 2	0.715*
Abortions (mean ± SD)	0.3 ± 0.6	0.5 ± 0.7	0.617*
Curettage procedures (mean ± SD)	0.3 ± 0.6	0.5 ± 0.7	0.617*
Previous cesarean sections (mean ± SD)	1.2 ± 0.8	0.5 ± 0.7	0.008*
No	2 (13.3)	13 (86.7)	< 0.001^#^
Yes	14 (63.6)	8 (36.4)	
1	10 (62.5)	6 (37.5)	
2	3 (60)	2 (40)	
3	1 (100)	0	

Abbreviation: SD, standard deviation.

Notes: *Mann-Whitney *U*-test; ^#^chi-squared test.

The mean gestational age at birth for women with placenta accreta was 35.4 ± 1.1 weeks. There was no need for maternal blood transfusion in any of the cases without placenta accreta; however, blood transfusion only occurred in one case of placenta accreta. This patient showed bladder invasion with resection of a bladder wall segment. There was no pelvic organ injury in any other case. The mean weight of the newborns was 2,635.9 ± 374.1 g, and the mean 1- and 5-minute Apgar scores were 7.9 ± 0.6 and 8.4 ± 0.6 respectively. There were four complications in the newborns in this group. Among the patients without placenta accreta, the mean gestational age was 35.6 ± 2.2 weeks, the mean birth weight was 2,486.9 ± 559.6 g, and the mean 1- and 5-minute Apgar scores were 6.7 ± 2.6 and 8.4 ± 0.9 respectively. There was one death and complications in nine newborns in this group (Table 2). All neonatal complications were infection or jaundice. A total of two neonates in the accretism group, as well as two neonates in the group without accretism, needed blood transfusions.

**Table 2:** Maternal and perinatal results of patients diagnosed with placenta previa with and without placenta accreta

Variable	With placenta accreta n = 16	Without placenta accreta n = 21	*p*-value
Gestational age at birth (mean ± SD), weeks	35.4 ± 1.1	35.6 ± 2.2	0.751*
Hysterectomy			
No	2 (9.5)	19 (90.5)	< 0.001^#^
Yes	14 (87.5)	2 (12.5)	
Maternal hospital stay after delivery (days)	3.4 ± 1.9	2.9 ± 2.2	0.735*
Birth weight (mean ± SD), grams	2,635.9 ± 374.1	2,486.9 ± 559.6	0.596*
1-minute Apgar score	7.9 ± 0.6	6.7 ± 2.6	0.280*
5-minute Apgar score	8.4 ± 0.6	8.4 ± 0.9	0.961*
Neonatal complications	4	9	0.123^#^
Neonatal hospital stay (days)	7.8 ± 3.1	8.2 ± 3.9	0.639*
Perinatal death	0	1	0.245^#^

Abbreviations: SD, standard deviation.

Notes: *Mann-Whitney *U*-test; ^#^chi-squared test.

Among the 37 (100%) women who underwent the US, the prevalence of placenta accreta was 20 (54.2%) (95% confidence interval [95%CI]: 40.8–67.3), and among the 21 (56.7%) who underwent the MRI, it was 66.7% (95%CI: 43.0–85.4). The measurements of the accuracy of the US and MRI examinations in the diagnosis of placenta accreta are presented in Table 3.

**Table 3:** Measurements of the accuracy of ultrasound and magnetic resonance imaging in the diagnosis of placenta accreta in pregnancies with placenta previa

Measurement	Ultrasound n = 37	Magnetic resonance imaging n = 21
Sensitivity (95%CI)	87.5 (71–96.5)	92.9 (66–99.8)
Specificity (95%CI)	44.4 (25.5–64.7)	42.9 (9.9–81.6)
Positive predictive value (95%CI)	65.1 (49.1–78.9)	76.5 (50.1–93.2)
Negative predictive value (95%CI)	75 (47.6–92.7)	75 (19.4–99.4)
Positive likelihood ratio (95%CI)	1.57 (1.10–2.26)	1.63 (0.84–3.10)
Negative likelihood ratio (95%CI)	0.28 (0.10–0.77)	0.17 (0.02–1.33)

Abbreviation: 95%CI, 95% confidence interval.

There was a prevalence of accretism in anterior versus posterior placentas according to the US exams (41.7%; 95% CI: 22.11–63.36 versus 46.2%; 95%CI: 19.22–74.87). The measurements of the diagnostic accuracy of the US and MRI at insertions of placenta are presented in Tables 4 and 5.

**Table 4:** Measurements of the accuracy of ultrasound and magnetic resonance imaging in the diagnosis of anterior placenta accreta in pregnancies with placenta previa

Measurement	Ultrasound n = 24	Magnetic resonance imaging n = 13
Sensitivity (95%CI)	100 (69.2–100)	100 (66.4–100)
Specificity (95%CI)	71.4 (41.9–91.6)	33.3 (4.3–77.7)
Positive predictive value (95%CI)	71.4 (41.9–91.6)	69.2 (38.5–90.9)
Negative predictive value (95%CI)	100 (69.2–100)	100 (15.8–100)
Positive likelihood ratio (95%CI)	3.5 (1.53–8.0)	1.5 (0.85–2.64)
Negative likelihood ratio (95%CI)	−	−

Abbreviation: 95%CI, 95% confidence interval.

**Table 5:** Measurements of the accuracy of ultrasound and magnetic resonance imaging in the diagnosis of posterior placenta accreta in pregnancies with placenta previa

Measurement	Ultrasound n = 13	Magnetic resonance imaging n = 8
Sensitivity (95%CI)	66.67 (22.3–95.7)	80 (28.4–99.5)
Specificity (95%CI)	100 (59.0–100)	100 (2.5–100)
Positive predictive value (95%CI)	100 (39.7–100)	100 (39.7–100)
Negative predictive value (95%CI)	77.8 (39.9–97.2)	50 (1.3–98.7)
Positive likelihood ratio (95%CI)	−	−
Negative likelihood ratio (95%CI)	0.33 (0.11–1.03)	0.20 (0.03–1.15)

Abbreviation: 95%CI, 95% confidence interval.

## Discussion

Placenta accreta is an obstetric condition; it can be fatal, and often requires a multidisciplinary approach.^11^ At our department, 100% of the patients with placenta accreta underwent C-sections, whereas 14 (87.5%) underwent total abdominal hysterectomy. In 2 (10%) cases, a point of cleavage was observed, and the placenta could be removed without the need for hysterectomy. This approach contributed to a very favorable outcome, that is, the absence of maternal mortality during the study period, and there was only one case of neonatal death.

A total of 11 (68.8%) out of the 16 patients with placenta accreta had anterior placenta, and 5 (31.2%) had posterior placenta. This finding corroborates with those of other studies, which reported a higher incidence of anterior placenta in confirmed cases of placenta accreta.^12^

The mean maternal age of the patients with placenta previa accreta was 31.8 years; this finding corroborates those found in the literature,^13^ ^14^ which show means of 31.6 and 32 years. Maternal age and number of children are also known to be associated with a high risk of placenta previa.^15^ The number of previous C-sections was higher in patients with placenta accreta, thus suggesting a strong relationship between prior uterine scars and the risk of invasion, a finding confirmed by other authors as well.^15^ However, this was not observed in relation to the mean number of pregnancies, deliveries, and abortions between the two groups. Our results do not corroborate the risk association reported in another study.^12^ Thus, because there was no significant statistical difference between the two groups, our study contradicts aspects that have already been reported in the reviewed literature.

No statistically significant differences were observed in the comparison of newborn parameters between the two groups (with and without placenta accreta); According to our current institutional protocol, when placenta accreta is diagnosed by imaging during antenatal care, the proposed surgical procedure is the classic uterine incision with fetal extraction followed by hysterectomy without attempting to remove the placenta from the uterine wall. Previously, each surgical approach was decided by the obstetrical team at the time of the C-section. In most cases, Pfannenstiel incisions in the skin, transverse incisions in the uterus, and attempts to remove the placenta were made. After this change in protocol, in addition to better neonatal well-being assessed by higher Apgar scores, we observed a marked decrease in the need for blood products for both the mother and the newborn.^16^

The consequences of a late diagnosis of placenta accreta can be severe; this emphasizes the importance of prior detection during antenatal care. The first and most crucial step must be early investigation, by asking women about their previous uterine surgeries and other possible factors related to placenta accreta, such as endometrial ablation or the use of assisted reproduction techniques.^17^ Women with myometrial damage primarily caused by previous C-sections are at an increased risk of developing placenta accreta.^11^

Antenatal diagnosis of placenta previa, placenta accreta, and its variants may reduce maternal and fetal morbidity and mortality, thus enabling the resolution of the pregnancy to be scheduled at tertiary institutions with multidisciplinary teams, neonatal intensive care units, and blood banks, among other resources available at the time of surgery. These measures can prove effective only if these conditions are created in advance, and if the correct diagnosis is made.^12^

The US remains the main imaging modality to screen for abnormal placental implantation; however, the MRI is also a very useful complementary imaging resource in cases of inconclusive US or posterior placenta.^18^ The MRI is clearly indicated when the US results in ambiguous conclusions.^19^ The accuracy of the US in the diagnosis of placenta accreta may be biased, as stated in a study^20^ in which a significant increase in the diagnostic accuracy was observed once the risk factors became known during the examination. In the same study,^20^ when a diverse group of patients with unknown obstetric history was assessed, the diagnostic performance characteristics of the US were shown to be less accurate.

A meta-analysis^21^ failed to show any significant difference between the US and the MRI for the diagnosis of placenta accreta. Both methods are highly specific and sensitive in diagnosing or ruling out the presence of placenta accreta, with the US being the first choice in patients with limited time and lower income.^21^ Another study^22^ also affirmed the good sensitivity of the US, but it showed that, although the MRI was not as useful as initially expected, it still provided additional information for women at risk. Another study^23^ has indicated the MRI as an excellent method for placenta assessment, particularly to investigate findings related to placental diseases, thus contributing to the adequate and timely care of pregnant women.^23^

Several examiners performed the US and MRI in the present study. The radiologists performing the MRI were blinded to the US results, except for the fact that the patients were diagnosed with placenta previa, and that the objective was to assess the presence and degree of placental invasion, which was one of the strengths of the study. However, during the US examinations, which were usually serial, the radiologists often became aware of the MRI results; this can be considered a limitation of the US assessment. This fact may not be relevant because almost 50% of the patients did not undergo an MRI; therefore, the examiners were blinded to the results.

The patient sampling started at the moment the MRI device was installed. During this period, the radiologists wished to evaluate all cases of placenta previa without prior knowledge of the US result in the presence or absence of placenta accreta, in an attempt to evaluate the previous experience of the service in identifying this finding. There were requests for MRI examinations; however, they were not performed in all cases due to the occasional technical unavailability of this examination in the institution.

The calculation of positive and negative predictive values depends on the prevalence of the disease; therefore, the prevalence for each diagnostic tool in the calculation of these measurements was taken into account. The prevalence of placenta accreta was identified separately for each diagnostic tool. The prevalence was higher using the MRI, but not due to the selection of cases. There were attempts to perform MRIs in all cases, but some patients were unable to undergo this examination.

The comparison of sensitivity and specificity between the US and the MRI in the diagnosis of placenta accreta indicated that the US had a good sensitivity, thus confirming the data from the literature. This eliminates the need for MRI in most cases, a factor that is particularly important in low-income countries with limited access to this examination.^18^

## Conclusion

In summary, regardless of the location of the placenta, the US and the MRI had similar sensitivity and specificity for the diagnosis of placenta accreta.
